# Nonlinear Pedagogy: An Effective Approach to Cater for Individual Differences in Learning a Sports Skill

**DOI:** 10.1371/journal.pone.0104744

**Published:** 2014-08-20

**Authors:** Miriam Chang Yi Lee, Jia Yi Chow, John Komar, Clara Wee Keat Tan, Chris Button

**Affiliations:** 1 Physical Education and Sports Science, National Institute of Education, Nanyang Technological University, Singapore, Singapore; 2 CETAPS, Faculty of Sports Sciences, University of Rouen, Rouen, France; 3 School of Physical Education, Sport and Exercise Sciences, University of Otago, Dunedin, New Zealand; University of Texas Health Science Center at San Antonio, Research Imaging Institute, United States of America

## Abstract

Learning a sports skill is a complex process in which practitioners are challenged to cater for individual differences. The main purpose of this study was to explore the effectiveness of a Nonlinear Pedagogy approach for learning a sports skill. Twenty-four 10-year-old females participated in a 4-week intervention involving either a Nonlinear Pedagogy (i.e.,manipulation of task constraints including equipment and rules) or a Linear Pedagogy (i.e., prescriptive, repetitive drills) approach to learn a tennis forehand stroke. Performance accuracy scores, movement criterion scores and kinematic data were measured during pre-intervention, post-intervention and retention tests. While both groups showed improvements in performance accuracy scores over time, the Nonlinear Pedagogy group displayed a greater number of movement clusters at post-test indicating the presence of degeneracy (i.e., many ways to achieve the same outcome). The results suggest that degeneracy is effective for learning a sports skill facilitated by a Nonlinear Pedagogy approach. These findings challenge the common misconception that there must be only one ideal movement solution for a task and thus have implications for coaches and educators when designing instructions for skill acquisition.

## Introduction

Motor skill acquisition during childhood forms the foundation for lifelong participation in sport, essential for long-term health and fitness benefits [1]. Learning a sports skill is a complex process that involves a multitude of factors. At the level of the learner, every individual is unique with differences in characteristics such as genetic composition, social-economic backgrounds, prior experiences, and learning styles [2]. A key challenge for movement practitioners is to cater for this abundance of individual characteristics during practice. Clearly, an instructional approach underpinned by a robust theoretical framework is essential for effective acquisition of a sports skill.

Traditionally, practitioners (e.g., coaches, teachers, etc.) have adopted approaches which are prescriptive and repetitive, utilizing technical demonstrations that provide learners with a “visual template or criterion model” for the desired skill [3]. The underlying assumption that has fuelled such pedagogies is that an ideal movement pattern exists for a task and that the practitioner's role is to help learners to recreate that pattern [3]. Furthermore, some theorists have suggested that learning is a gradual, linear process offering the ‘power law’ as supportive evidence [4], [5], although this viewpoint is not without its critics [6]. An increasing amount of evidence from the Dynamical Systems Theory (DST) perspective challenge these traditional assumptions about skill acquisition [3], [7], [8].

From the DST viewpoint, the learning process does not generally follow continuous linear progressions of behaviour but rather involves sudden discontinuous changes over time [9]. Learners should be conceived as nonlinear dynamical systems, comprising numerous component parts that interact and self-organize to form stable patterns. The emergence of self-organized functional movement solutions is faciliated through the interaction of performer, task and environment constraints which act as boundaries to shape goal-directed behaviours. Unlike tradtional approaches in motor learning, there is no central controller (e.g., an authoritative coach) that determines how movement behaviors should be produced to meet task goals [7]. Chow et al. [10] were the first to propose the use of the umbrella term “Nonlinear Pedagogy” to capture how practitioners can benefit from these contemporary theoretical concepts.

Grounded in the constraints-led approach, Nonlinear Pedagogy (referred to from here as NP) advocates the manipulation of key constraints that form boundaries for the learner to explore functional movement solutions [11], [12]. Thus, NP provides a framework for pedagocial principles that can be used to account for the nonlinear behavioural changes typically observed in learning movement skills. It provides a theoretical impetus for practitioners to incorporate “representativeness, manipulation of constraints, attentional focus, functional variability, and the maintenance of pertinent information-movement couplings” to effectively design motor learning interventions [13]. Firstly, NP emphasizes the importance of a representative learning design in which learning is situated in real-game contexts. When placed in representative learning environments, information-movement couplings relevant to the desired sports skill are presented to the learner, providing functional affordances (i.e., opportunities for action) for the individual. NP also advocates the manipulation of task constraints such as instructions, rules of the activity, and equipment (e.g., racquets, balls, court size), to encourage learners to explore various movements solutions most suitable for themselves. In addition, just as instructions based on external focus of attention seem to reduce the conscious processing of information in skill acquisition [14], NP focuses on movement outcomes rather than movement form. At this juncture, it is important to highlight that while the NP approach may encompass practical features similar to external focus of attention, NP is underpinned by key aspects from DST and thus the guiding principles for implementation of the two approaches are not the same. Lastly, NP embraces functional movement variability in developing a coordinated movement, since movement variability is a critical feature of learning in human movement systems.

When it comes to teaching sports skills, NP provides the appropriate framework for practitioners to cater for individual complexities and dynamic learning environments. In NP, learners are encouraged to experiment with different movement patterns and adapt individual coordinative structures to achieve functional movement solutions [15]. This process, known as degeneracy, is crucial in skill acquisition as it empowers the individual with a variety of movement possibilities that may be recruited to suit task and environmental demands. According to Bernstein [16], learners form functional muscle-joint linkages, known as coordinative structures, to deal with the numerous component parts present in the human movement system. Every time a new movement task is performed, these coordinative structures are reassembled temporarily and flexibility, adapting the various component parts to suit the specific task condition [17]. Degeneracy is the ability for complex neurobiological systems (i.e., learners in this case) to achieve different solutions for the same task goals and provides the individual with an improved capacity to deal with information-rich dynamic environments [7], [18]. Previous studies have shown that there are individual differences in the acquisition of coordination and control even when presented with the same task [19], [20].

It seems reasonable to expect that the NP approach would produce a greater variety of movement solutions compared to the traditional instructional approach for acquisition of sports skills. Traditional motor learning pedagogies involve instructions that are prescriptive, repetitive, and drill-like with a strong focus on “criterion model” technique, labelled as Linear Pedagogy (LP) in this study. While learners taught with a LP approach are expected to reproduce movement patterns that are more similar to the “criterion model”, the NP approach is likely to produce a greater variety of movement patterns that are more suited to the individual. A recent study provides initial evidence that learners taught with a NP approach were able to develop functional ‘personal patterns of coordination’ which were more biomechanically efficient in breastroke swimming indicated by a longer gliding time and a coordination pattern closer to anti-phase with learning [21]. There are also examples in the literature to indicate that explorative instructions (e.g., analogy, external focus of attention and manipulation of task constraints) tend to produce better results than instructions that are generally prescriptive and drill-like [14], [22], [23]. These studies suggest that explorative instructions, which contain features of NP, could result in greater performance benefits although more evidence is still required.

It is unclear how a NP approach adopted within a controlled school setting will influence motor skill acquisition. The purpose of this study was to investigate the effectiveness of a NP approach, in relation to a LP approach. It was predicted that: 1) The LP group would produce higher movement criterion scores, indicating a movement pattern that is more similar to the “criterion model”; 2) The NP group would result in a greater number of movement clusters, indicating the presence of degeneracy; 3) Both groups would improve in performance accuracy scores although the NP group would produce higher scores as a result of the expression of degenerate behavior.

## Methods

### Participants

24 right-handed female participants aged 9 to 10 years were selected for this study and randomly assigned to either the NP or the LP group. Three participants did not complete the study due to various reasons, leaving 11 participants in the NP group (mean weight = 31.33±2.72 kg; mean height = 133.00±2.30 cm) and 10 participants in the LP group (mean weight = 31.97±2.16 kg; mean height = 140.70±2.17 cm). All participants were novices with limited experience in playing any racquet sports. Prior to the start of the study, written informed parental consent and participant assent was obtained. Ethics approval was granted by the Nanyang Technological University Institutional Review Board.

### Task

Participants within each experimental group were required to learn the tennis forehand groundstroke in a modified tennis setting. The task involved a modified tennis ball being played to the participants by the experimenter. The goal was to return the ball using a forehand groundstroke over the net to several pre-assigned targets on the court.

### Procedures

The present study consisted of a pre-intervention session, followed by a 4-week intervention (eight sessions, each lasting 15 minutes), a post-intervention session and a retention session conducted four weeks after the intervention.

#### 1. Testing procedures

The pre, post and retention sessions comprised the same procedures administered at the respective time points of the study. At the start of the testing sessions, a demonstration video of the experimental task was shown to participants. Following this, reflective markers were placed on 17 anatomical landmarks namely: the acromion process, medial and lateral epicondyles, medial and lateral styliod process, illac crests, and greater trochanter on the left and right sides, as well as three markers on the left upper arm, right forearm, and right upper back to assist in identification of the left and right segments [24]. Five reflective markers were also placed at positions corresponding to 2-, 3-, 4-, 8-, and 10'o-clock on the racquet face to identify the racquet head segment. The markers were required to build a 3D-model of the body segments and racquet to obtain kinematic movement data of each participant.

**Figure 1:**
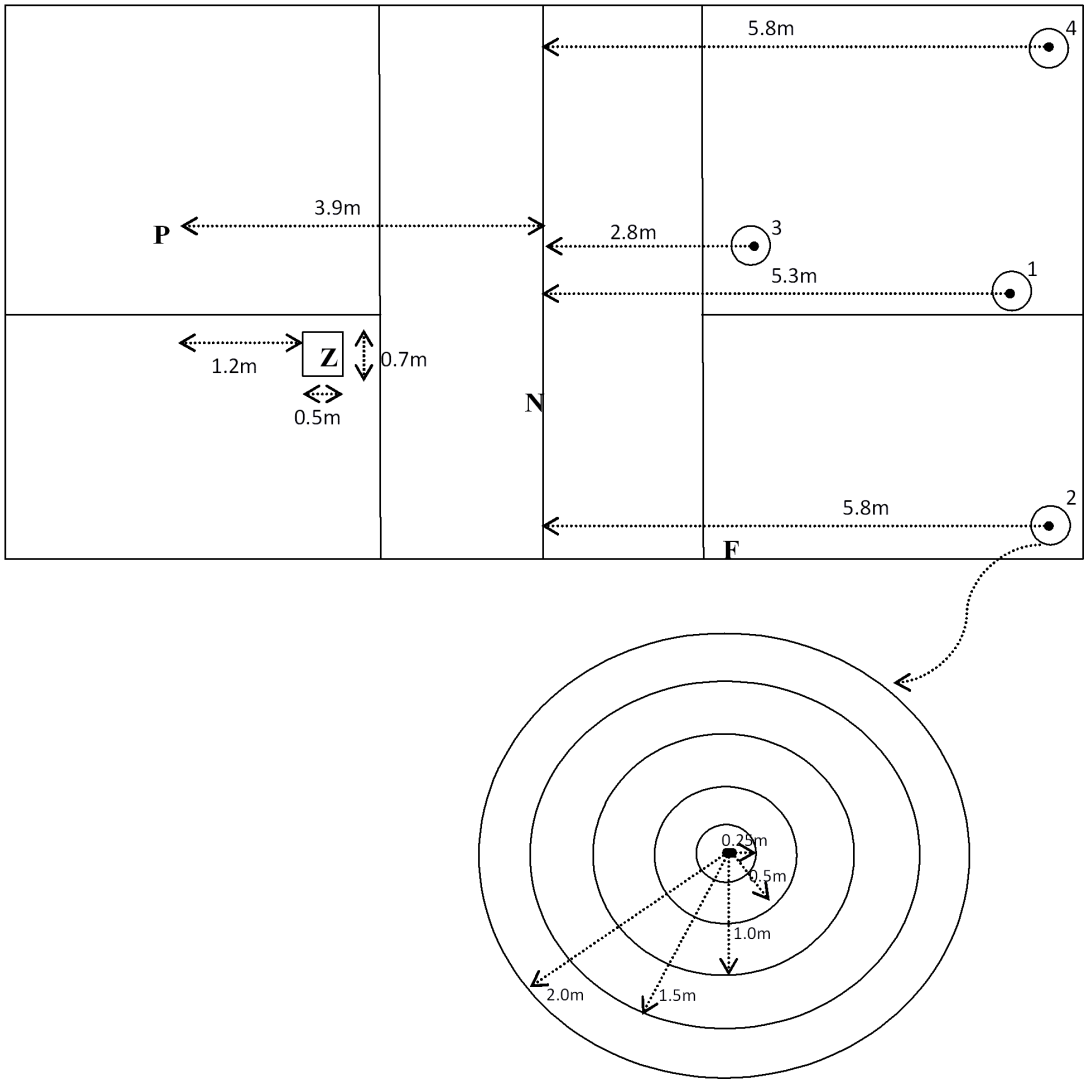
Schematic diagram of test set-up. Figure includes position of participant (**P**) in relation to feeding zone (**Z**), net (**N**), target zones (1, 2, 3 and 4) and feeder (**F**) as well as dimensions of feeding zone and target zones.

After 10 warm-up trials consisting of hitting over the net (0.8 m) with no accuracy demands, participants performed 10 forehand trials aimed at a target on the court (target 1; referred to as specific target trials) followed by 15 forehand trials aimed at three different targets (target 2, 3 and 4; referred to as multiple target trials) requiring them to vary their shots in a pre-assigned sequence (See Figure 1). During these trials, a modified-tennis ball (Gamma foam ball) was played by the feeder to land in front of the participants. A live feeder was preferred over a machine feed as it allowed participants to utilize the cues from the movement of the racquet, similar to returning a tennis shot in an authentic tennis game [25]. To ensure that the feed was consistent, the ball had to land within a marked out feeding zone (0.7 m×0.5 m). A trial in which the feed was not of acceptable accuracy was repeated and the consistency of the feed was later validated by an expert tennis coach.

#### 2. Interventions

Following the pre-test, the participants underwent a 4-week practice intervention, with 15 minute lessons twice a week, to learn a forehand groundstroke taught using either the NP or LP approach. For both the NP and LP groups, each practice session consisted of 80 forehand groundstroke trials with instructions from either the NP or LP approach. The volume of practice for both groups was comparable with a total of 120 minutes of practice over 4 weeks comprising 640 practice trials. The intervention was developed by the research team and verified by two academics knowledgeable in the area of NP and LP approaches respectively, but external to this research. Both NP and LP interventions were delivered by the same researcher according to the instructions and activity plans developed prior to the intervention phase.

The NP and LP interventions were designed on the understanding that learners could be viewed as either nonlinear or linear systems respectively. The key differences between the nonlinear and linear systems are explained as follows (for detailed justification, see Chow et al. [26]). Firstly, small changes to practice task constraints, such as instructions or equipment, may lead to non-proportionate changes in performance for nonlinear systems, while in linear systems it is assumed that a change in practice task leads to a proportionate change in performance. Another difference describes how nonlinear systems are multi-stable (i.e., one cause may result in multiple possible behavioral effects) whereas linear systems are mono-stable (i.e., one cause will result in a single behavioral effect). Multi-stability can be facilitated through the manipulation of a relevant task constraint in NP. Parametric control is the third characteristic of nonlinear systems whereas linear systems involve a centralized control (e.g., top-down, prescriptive approach in LP). Lastly, variability or noise can play a functional role in the likelihood of nonlinear systems transiting between multiple states (i.e., exploration of multiple solutions) whereas in linear systems, noise is seen to play a detrimental role and produces undesired output variability.

Essentially, NP addresses the nonlinearity in learners through the manipulation of constraints, mainly task constraints, creating boundaries for the learner to explore and discover functional movement solutions. The manipulation of task constraints in the NP condition included the manipulation of net height, target area, court size, and rules to achieve specific task goals. In addition, instructions were outcome-focused such as “Hit ball flight like shape of a rainbow”; “Call out color and hit to zone”; “Hit to baseline zone”. These conditions also encouraged functional variability such as the use of a variety of balls and racquets, and hitting to different target zones. An important attribute of the NP approach was the application of Newell's [27] stages of learning (Coordination, Control, and Skill) that focuses on individual learning rates for learning a movement skill. Within each stage of learning, the milestones for teaching net-barrier games were adapted from Hopper [28]: ‘consistency’ (contact with ball, hit over net, hit within opposite court), ‘accuracy’ (hit to target), ‘depth’ (hit to baseline), and ‘spin’ (develop topspin). Participants from this group were provided with instructions according to their individual progression which was assessed during the last 10 trials of each session. If a participant achieved a score of 8 or more, determined from pilot sessions, the participant progressed to the next milestone.

In contrast, LP comes from a traditional perspective that views learners as linear systems and that all learners should aspire towards a common, idealized motor pattern. LP instructions involved a “centralized control” where practitioners instruct what is considered as the ideal movement pattern for a task through the use of prescriptive cues and repetitive drills, leaving negligible opportunity for exploration (see Chow [13] for brief discussion on NP and traditional pedagogical approaches that typifies ‘LP’). The LP condition in this study consisted of prescriptive tennis forehand groundstroke technique instructions and repetitive practice drills. The forehand cues provided were according to the different phases of a forehand stroke: 1) Preparation: “Shake hands with racquet”, “Racquet in front, feet shoulder-width apart”; 2) Pre-execution: “Turn shoulders to side and bring racquet back”, “Move to ball and step”; 3) Execution: “Moving racquet low to high, contact ball in front of forward foot”; 4) Follow through: “Follow through and end swing above opposite shoulder” [29]. The tennis cues, which are common in a traditional tennis lesson, were developed and verified by an expert tennis coach who was blinded to the purpose of this study. At the start of the practice session and at intervals of every 15 trials, the forehand stroke was demonstrated according to the cues and participants had to perform a shadow drill following the experimenter.

### Apparatus and measurements

#### 1. Performance accuracy scores

Performance accuracy scores were determined based on a 6-point rating scale according to the landing position of the ball for the assigned target of the respective trial (6 points = on target; 4 to 1 points given according to distance away from center of target; 0 = out of target). The suitability of the position of the target zone and the scoring system was confirmed during pilot sessions prior to the study. Such scoring scales were also used in previous studies to investigate learning a sports skill in soccer kicking [19] and basketball shooting [30].

#### 2. Movement criterion scores

Movement performance of the forehand groundstroke were captured by a digital video (DV) camera (Canon, Legria HF S100) positioned approximately 5 m to the right side of the participant and fixed to a 1.2 m high tripod. Qualitative forehand movement performance was rated according to a movement checklist which consisted of the critical features of a traditional tennis forehand stroke [29]. The critical features on the movement checklist were validated by an expert tennis coach. The movement performance was rated for the 10 specific and 15 multiple target trials in each test session, according to the frequency each critical feature was observed (0 = never; 1 = sometimes; 2 = always). A total score (maximum score = 20) was given for the specific and multiple target trials respectively for each participant, with a higher score indicating a movement pattern to be more similar to the “criterion model” of the forehand stroke. All trials were rated by a trained research assistant who was blinded to the study. Intraclass correlation coefficient (ICC = 0.938) of forty randomly selected trials rated by an expert tennis coach and the research assistant indicated a high reliability for the movement criterion score ratings.

#### 3. Kinematic movement pattern

The 22 spherical reflective markers (19 mm) placed on the assigned landmarks were captured by 7 infrared cameras (Hawk Digital Camera, Motion Analysis Corporation) and recorded at 200 Hz on the cortex software (Motion Analysis Corporation, SantaRosa, CA, USA). Capture of the reflective markers enabled a 3D model for each participant to be built and subsequently the kinematic data for the relevant joint angles to be determined. The displacement of the markers in 3D space was processed using the cortex software for all 10 specific target trials pre, post and retention tests. After visual inspection, a low pass Butterworth digital filter at a frequency of 10 Hz was applied [31]. Visual 3D software (C-Motion Inc) was then used to construct a 6-segment upper body model (upper arm, forearm, thorax and pelvis) and the racquet head segment. 3D kinematic variables and joint angles were calculated from the respective segments for each individual participant.

Based on previous tennis studies examining the forehand tennis strokes, 12 time-continuous kinematics variables were chosen for further analysis: Right and left elbow flexion/extension, right and left shoulder flexion/extension, abduction/adduction and internal/external rotation, thorax and pelvis rotation, separation angle (difference between hip and shoulder rotation angle) and racquet rotation angle (information about racquet horizontal displacement in the transverse plane) [32], [33], [34]. The ‘start’ of each forehand swing was defined as the point preceding any backward movement of the racquet in the y-direction (front/back direction) and the ‘end’ was determined as the furthest racquet horizontal displacement in the transverse plane. All trials were normalized to 100 data points between the ‘start’ to the ‘end’ for simultaneous comparison across trials and individuals [35]. The normalized time-continuous kinematic data was subsequently used for cluster analysis (see section on ‘Inter-individual cluster analysis’).

### Data analysis

#### 1. Statistical analysis (performance accuracy and movement criterion scores)

This study followed a 3 (time: pre, post, retention)×2 (intervention: NP and LP group) factorial design. A mixed-design ANOVA was used to determine differences within and between groups for two dependent variables: performance accuracy scores and movement criterion scores. Any violation in sphericity was corrected using Greenhouse-Geisser epsilon (ε). Fishers LSD *post hoc* test was used to further analyze significant main effects and interactions to determine the location of differences within (time) and between (intervention) factors. Statistical difference was accepted at *p*<0.05 and effect size was calculated using partial eta squared (η_p_^2^).

#### 2. Inter-individual cluster analysis (movement clusters)

Cluster analysis is typically used to examine changes in movement patterns and to determine the presence of movement variability. In particular, cluster analysis is used to group individual trials based on kinematic or kinetic variables (rather than performance scores) so that natural groupings can be observed in the data [36]. According to Rein et al. [37] cluster analysis invovles three main steps: 1) Data pre-processing (selection and processing of appropriate kinematic variables), 2) Cluster analysis, and 3) Cluster validation. The application of cluster analysis methods has been commonly used to examine movement pattern variability in variety of movement skills such as swimming [38], walking [39], kicking [40] and throwing [41]. Therefore, cluster analysis was deemed suitable as no *a priori* knowledge was available on possible groupings between individuals or trials.

In this study, cluster analysis was used to identify the presence of degeneracy for learning a tennis forehand stroke. Specifically, cluster analysis determined the number of movement clusters found in each group for specific target trials following procedures used by Komar and colleagues [38]. A greater number of movement clusters indicated the presence of degeneracy in a given data set. It was expected that the NP approach would produce a greater number of movement clusters following the intervention as this approach facilitated exploration, while the LP approach was likely to display fewer movement cluster as all participants were taught to conform to an idealized motor pattern.

The 12 time-continuous kinematics variables of participants were selected as input variables into a cluster analysis using the Fisher-EM algorithm [42]. This kind of algorithm is based on a probabilistic model which projects the data in a latent subspace at each iteration in such a way that emerging clusters maximize the Fisher information (i.e. maximize the inter-cluster distance while minimizing the intra-cluster distance). Cluster analysis was run for each respective test session (pre, post or retention), for each intervention group (NP or LP) seperately (i.e., six data sets). Each data set comprised 100 trials (10 participants×10 trials). Subsequently, the Bayesian Information Criterion (BIC) index was used as a model-selection criterion to validate the number of clusters found in each respectively data set. Cluster analysis was performed for a potential number of cluster from 2 to 12 and represented in a vector containing the BIC index (×10^5^) for [2], [3], [4], [5], [6], [7], [8], [9], [10], [11], [12] number of clusters. The number of clusters which corresponded to the first local maximum represented the ideal number of clusters that best fitted the data set (i.e., that shows the highest ratio between inter-cluster distance and intra-cluster distance) [43].

In addition to the Fisher-EM algorithm, the dimension of the latent subspace where the clustering was performed could be modulated by a “sparsity” parameter. The sparsity index which corresponded to the “weight” of each point into the clustering was also calculated for each kinematic variable. The average sparsity index for each kinematic variable (i.e., from 100 data points) indicated which variable was most discriminative. A higher sparsity value indicated that the respective kinematic variable had a higher percentage contribution to the clustering. Mean movement patterns for kinematic variables, selected based on sparsity values, were displayed on angle-plots for each movement cluster. Movement clusters were indicated as C1…C7 for each respective data set. The mean movement pattern of each cluster was determined from the kinematic data of the trials assigned to a particular cluster. The number of trials and distribution of participants found in each cluster was also displayed in a bar chart to examine the changes in the composition of the movement clusters over time.

## Results

### Performance accuracy scores

For specific target trials, a main effect for time was observed, F (2,38) = 3.386, *p* = 0.044, η_p_^2^ = 0.151. *Post hoc* analysis showed that performance accuracy scores were significantly different (p = 0.019) from pre (NP = 0.62±0.53; LP = 0.65±0.59) to post test (NP = 0.95±0.44; LP = 1.10±0.78). A main effect for time was also observed for multiple target trials F (2,38) = 3.616, *p* = 0.037, η_p_^2^ = 0.160. *Post hoc* analysis showed that performance accuracy scores were significantly different (*p* = 0.013) from pre (NP = 0.56±0.56; LP = 0.68±0.39) to retention test (NP = 1.05±0.49; LP = 0.93±0.72). The time×intervention interaction was not significant for specific (F(2, 38) = 0.079, *p*>0.05, η_p_^2^ = 0.004) and multiple trials (F(2, 38) = 0.600, p>0.05, η_p_^2^ = 0.031). In addition, no significant differences (*p*>0.05) were observed between the intervention groups for both specific and multiple target trials.

### Movement criterion scores

**Figure 2:**
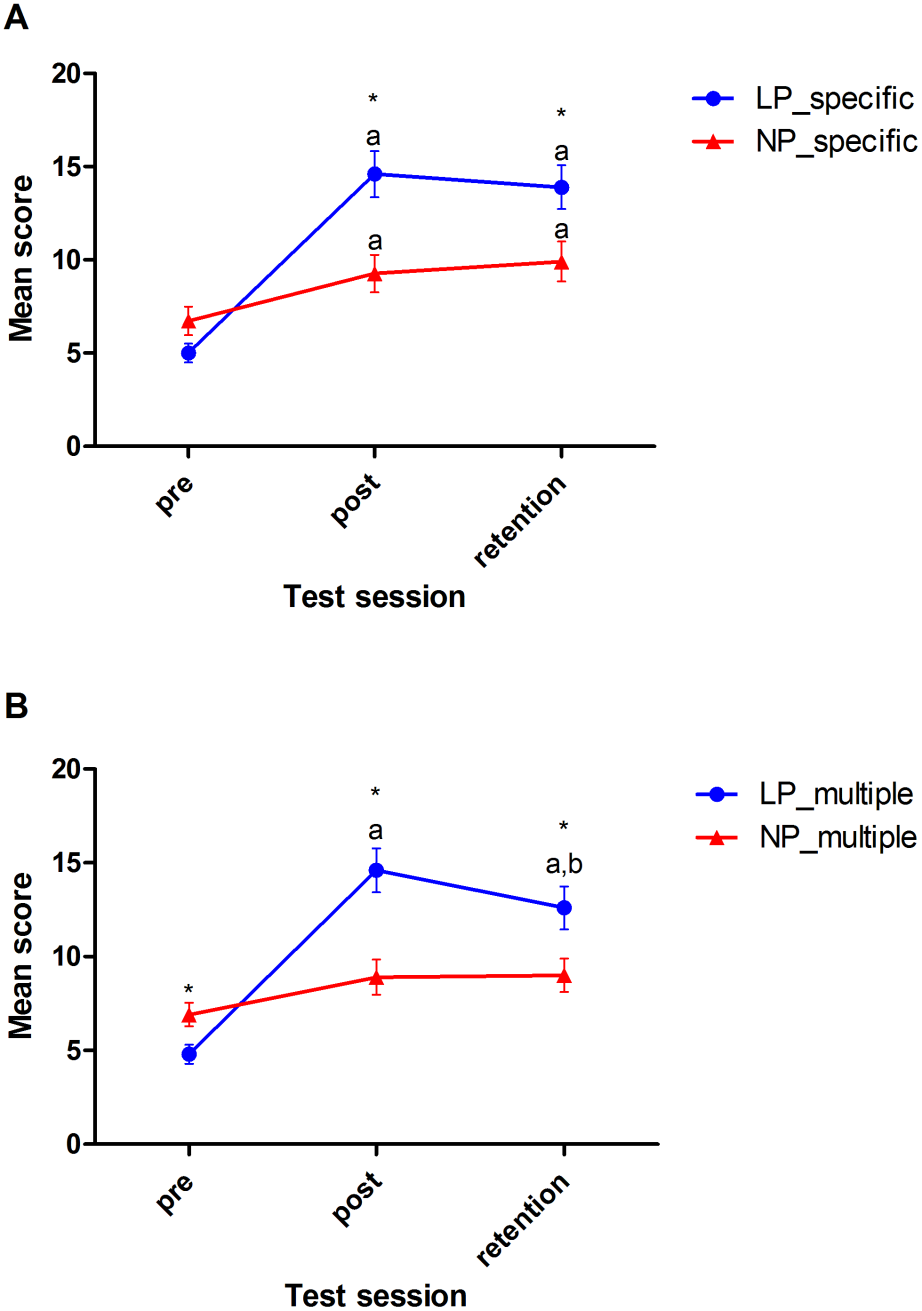
Mean movement criterion scores for (A) specific and (B) multiple target trials at pre, post and retention tests. (a) indicates significant differences from pre-test (*p*<0.05), (b) indicates significant differences from post-test (*p*<0.05) and (*) indicates significant differences between groups (*p*<0.05). Error bars represent Standard Error Measure (S.E.M) (n = 21).

For specific target trials, mean movement criterion scores showed a main effect for time (F(2,38) = 42.559, *p*<0.001, η_p_^2^ = 0.691) and intervention (F(1,19) = 5.313, *p*<0.001, η_p_^2^ = 0.219), with a significant time×intervention interaction (2,38) = 12.217, *p*<0.001, η_p_^2^ = 0.391). Figure 2A shows the nature of the interaction effect. *Post hoc* analysis showed that both the NP and LP group increased significantly in movement criterion scores from pre to post (LP: *p*<0.001; NP: *p* = 0.049) and from pre to retention (LP: *p*<0.001; NP: *p* = 0.012), with significantly higher scores for the LP group at post (NP = 9.27±3.35; LP = 14.60±3.92; *p* = 0.003) and retention tests (NP = 9.91±3.53; LP = 13.90±3.73; *p* = 0.021).

For multiple target trials, mean movement criterion scores showed a main effect for time (F(1.20,22.73) = 37.845, *p*<0.001, η_p_^2^ = 0.666, ε = 0.598) and intervention (F(1,19) = 6.085, *p* = 0.023, η_p_^2^ = 0.243), with a significant time×intervention interaction (F(1.20,22.73) = 15.377, *p*<0.001, η_p_^2^ = 0.447, ε = 0.598). Figure 2B shows the nature of the interaction effect. *Post hoc* analysis showed that movement criterion scores changed only for the LP group with a significant increase from pre to post (*p*<0.001) and from pre to retention test (*p*<0.001), and a significant decrease from post to retention test (*p*<0.001). In addition, the movement criterion scores were significantly higher for the NP group at pre-test (NP = 6.91±2.071; LP = 4.80±1.62; *p* = 0.018), but resulted in significantly higher scores for the LP group at post (NP = 8.91±3.11; LP = 14.60±3.72; *p* = 0.001) and retention tests (NP = 9.00±2.97; LP = 12.60±3.60; *p* = 0.021).

### Movement clusters

One participant in the NP group was excluded from the movement cluster analysis due to issues with the calibration during data collection, thus only 10 participants from each group were included for cluster analysis.

#### 1. Cluster validation

Fisher-EM clustering for the kinematic variables at pre-test showed that the first local maximum of the BIC index corresponded with 4 clusters for both NP (BIC index (×10^5^) = [−5.19, −5.16, ***−5.14***, −5.14, −5.13, −5.18, −5.15, −5.20, −5.19, −5.20, −5.22]) and LP (BIC index (×10^5^) = [−5.21, −5.17, ***−5.13***, −5.14, −5.12, −5.13, −5.17, −5.17, −5.18, −5.18, −5.22]). In addition, the first local maximum of the BIC index corresponded with 7 clusters for NP (BIC index (×10^5^) = [−5.31, −5.29, −5.27, −5.25, −5.25, ***−5.24***, −5.28, −5.28, −5.29, −5.32, −5.34]) and 3 clusters for LP (BIC index (×10^5^) = [−5.33, ***−5.28***, −5.30, −5.27, −5.26, −5.26, −5.26, −5.26, −5.30, −5.31, −5.32]) at post-test, followed by 5 clusters for NP (BIC index (×10^5^) = [−5.28, −5.26, −5.24, *−* ***5.21***, −5.22, −5.23, −5.24, −5.26, −5.27, −5.27, −5.31]) and 7 clusters for LP (BIC index (×10^5^) = [−5.32, −5.27, −5.26, −5.25, −5.24, ***−5.23***, −5.27, −5.28, −5.26, −5.27, −5.30]) at retention test.

The higher sparsity values indicate that the respective kinematic variable represents a higher percentage contribution to the clustering. Right and left elbow flexion/extension, right and left shoulder internal/external rotation, and racquet rotation angle were the five kinematic variables with higher sparsity values (Table 1). From here, two of the kinematic variables were selected for examination of the mean movement patterns to discriminate the movement clusters. The right shoulder rotation was selected as it had the highest sparsity among the right-sided joint angles and racquet rotation angle was chosen as it provided information about the racquet position.

**Table 1:** Mean sparsity index (%) for each kinematic variable.

	NP				LP			
	Pre	post	ret	Avg^a^	pre	post	Ret	Avg^a^
Right elbow (flex/ext)	7.44	8.29	8.56	**8.10**	8.69	9.08	7.22	**8.33**
Left elbow (flex/ext)	7.28	8.38	9.23	**8.30**	10.01	11.71	8.51	**10.08**
Right shoulder (flex/ext)	6.05	6.86	6.89	6.60	6.46	4.11	8.86	6.48
Right shoulder (abd/add)	6.12	7.68	6.58	6.79	8.49	5.95	7.54	7.33
Right shoulder (int/ext rotation)	13.70	13.89	14.69	**14.09**	12.92	13.99	16.31	**14.41**
Left shoulder (flex/ext)	5.79	6.48	7.62	6.63	6.22	6.99	7.12	6.78
Left shoulder (abd/add)	6.65	6.77	8.80	7.40	6.38	6.70	5.87	6.32
Left shoulder (int/ext rotation)	15.77	14.39	12.56	**14.24**	15.13	16.62	12.76	**14.84**
Separation angle	6.23	4.93	4.33	5.16	4.55	3.10	5.83	4.49
Thorax rotation	5.86	5.62	5.88	5.78	5.00	7.32	4.89	5.74
Pelvis rotation	6.72	5.78	5.56	6.02	4.59	5.46	5.70	5.25
Racquet rotation angle	12.41	10.94	9.31	**10.88**	11.57	8.95	9.39	**9.97**
Total^b^	100	100	100	100	100	100	100	100

a Refers to the average of the mean sparsity (%) for each kinematic variable for the NP and LP group.

b Refers to the sum of all 12 kinematic variables' mean sparsity (%) for each test session.

#### 2. Mean movement patterns for each cluster

**Figure 3:**
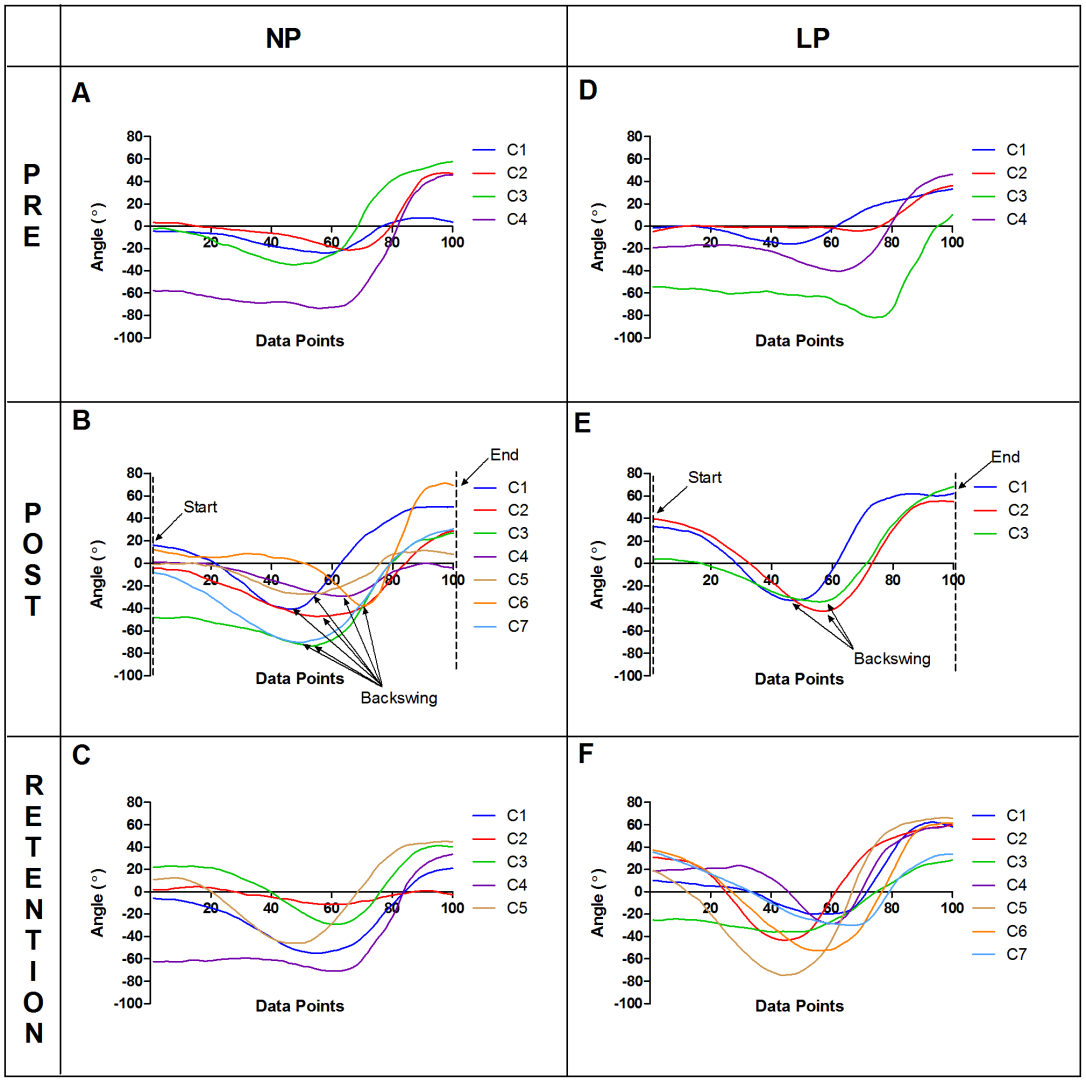
Mean right shoulder internal/external rotation angle. Mean kinematic data normalized to 100 data points for movement clusters found in specific target trials for the six data sets: NP - pre (A), post (B), retention (C); LP - pre (D), post (E), retention (F). C1, C2…C7 represent movement clusters found within each data set. Positive angles indicate internal rotation and negative angles indicate external rotation. The “Backswing” is approximated based on the point where the external rotation was the greatest.

**Figure 4:**
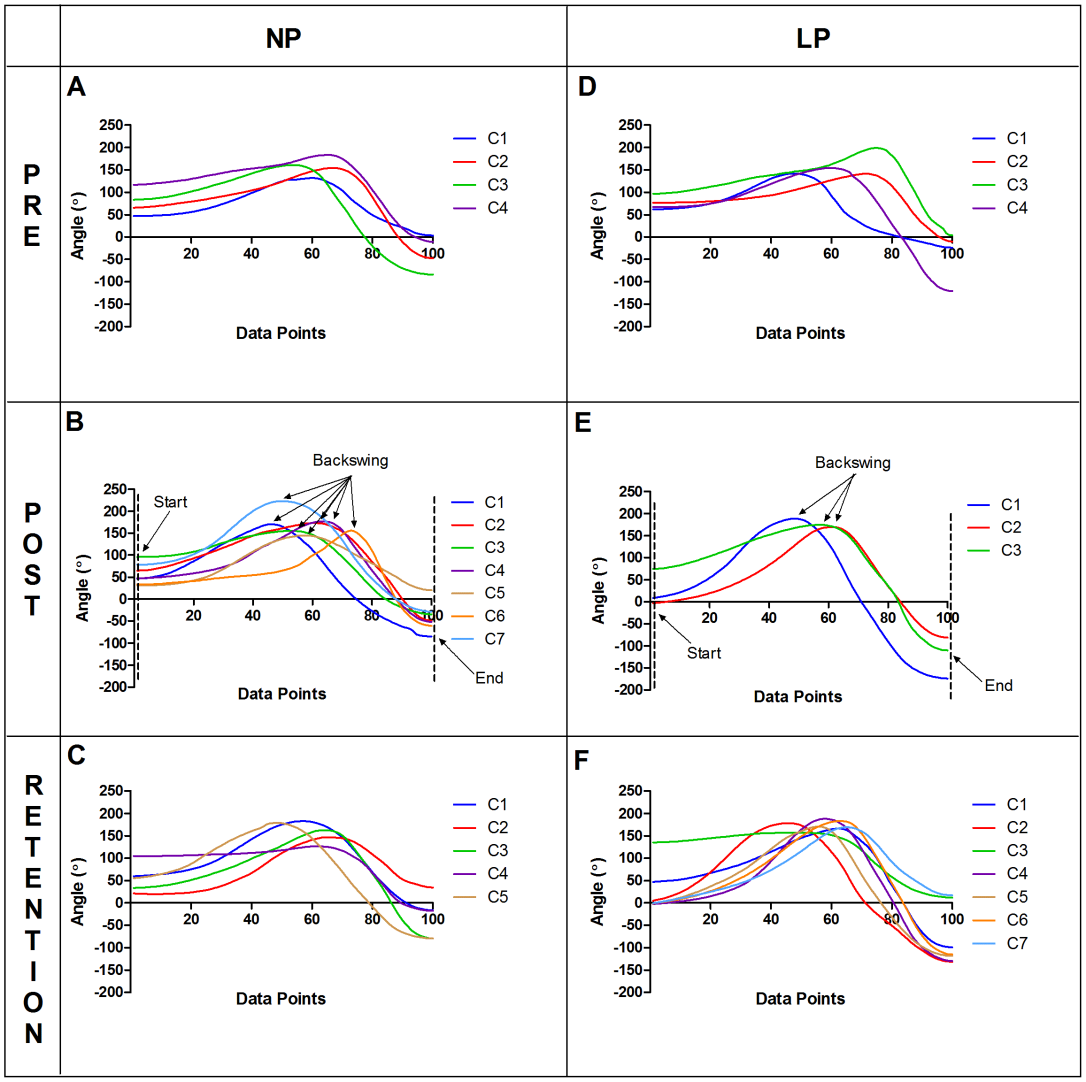
Mean racquet rotation angle. Mean kinematic data normalized to 100 data points for movement clusters found in specific target trials for the six data sets: NP - pre (A), post (B), retention (C); LP - pre (D), post (E), retention (F). C1, C2…C7 represent movement clusters found within each data set. An angle of 0° indicated that the racquet was pointing towards the net while an angle of 180° indicated that the racquet was pointing towards the baseline (typically at or near backswing). The “Backswing” is approximated based on the point where the racquet rotation was the greatest.

The movement patterns within each group showed changes following the 4-week intervention, although both groups changed in different ways. At post-test, the LP group displayed movement patterns that were relatively uniform across all participants in C1 to C3 (i.e., cluster 1 to cluster 3). For example, Figure 3E shows that the LP group generally displayed consistent patterns for shoulder rotation at post-test: internal rotation at the start of the swing (preparation phase), followed by external rotation at backswing and internal rotation at the end of the swing (follow through). Figure 4E shows that for the LP group at post-test, both C1 and C2 exhibited a racquet rotation angle close to 0° at the start of the swing (preperation phase), followed by C1 to C3 displaying angles close to 180° at backswing, ending with angles close to −90° and below at the end of swing (follow through). On the other hand,the NP group did not show such consistency but displayed greater variabilty from cluster to cluster in terms of the general movement pattern and the range of motion of the forehand swing at post-test. For example, the angles that occurred at backswing and at the end of the swing varied from C1 to C7 as seen in Figure 3B and 4B.

At retention test, the movement patterns in the LP group changed drastically displaying a greater variety of kinematic movement patterns for the forehand stroke (Figure 3F and 4F). The NP group continued to display a variety of movement patterns at retention test as seen in Figure 3C and 4C.

#### 3. Distribution of trials across clusters

**Figure 5:**
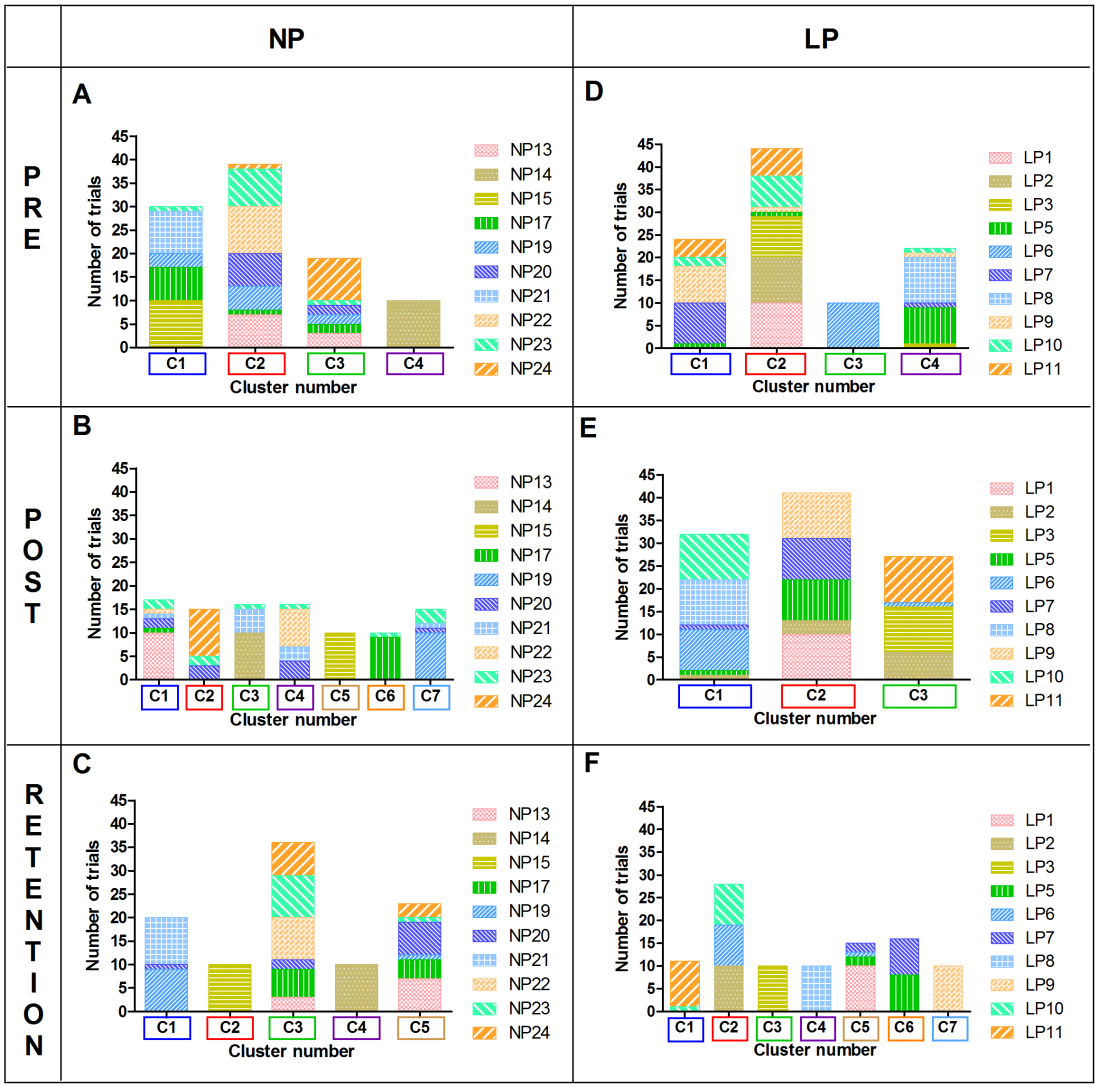
Number of trials and distribution of participants in each cluster. Each bar represents the number of trials and distribution of participants found in each movement cluster for specific target trials in the six data sets: NP - pre (A), post (B), retention (C); LP - pre (D), post (E), retention (F). C1, C2…C7 represent movement clusters found within each data set. Participants are labelled as LP1…LP11 (for LP group) and NP13…NP24 (for NP group).

Each movement cluster in Figure 5 corresponded to the movement cluster in Figure 3 and 4 for each respective data set. For example, C1 in Figure 5A comprised 30 trials from which the mean movement pattern was determined for C1 in Figure 3A and 4A. Figure 5A and 5D shows that the distribution of trials in the movement clusters was similar in both NP and LP group at pre-test. For example, the cluster with the most number of trials at pre-test (C2 in Figure 5A and Figure 5D), consisted of 39 and 44 trials from seven participants for NP and LP groups respectively, while the smallest cluster (C4 in NP and C3 in LP) only comprised 10 trials from one participant. Following the intervention, the number of trials and distribution of participants in each cluster changed considerablly at post-test. The distribution of participants across the clusters was more varied in the NP group with some of the same partcipants distributed across several clusters (Figure 5B). In the LP group, most of the participants were found in C1 and C2 with the highest number of the trials assigned to C2 (41 trials) followed by C1 (32 trials) (Figure 5E). At retention test, the distribution of trials changed again especially for the LP group in which partiticipants were spread throughout the 7 clusters (Figure 5F).

## Discussion

The purpose of this study was to investigate the effectiveness of a NP approach for learning a tennis forehand stroke in a controlled setting. Based on previous literature, it was predicted that while the NP group would perform better in accuracy scores, it would produce lower movement criterion scores and display a greater variety of movement patterns to achieve the task goal, as compared to the LP group, indicating the presence of degeneracy.

Following the 4-week intervention, both NP and LP groups improved in performance accuracy scores just as well. Improvements in accuracy scores for the NP group occurred despite an intervention that encouraged learners to explore functional movement solutions, without involving prescriptive, repetitive instructions. Perhaps the NP approach required a longer intervention time to be most effective and for the children to discover more fully the functional movement solutions for themselves. Even though the initial prediction was not met, the results provide evidence that it is not necessary for learners to achieve the “criterion model” in order to be successful and that the NP approach, which encourages exploration and variability, is effective [3], [19].

Information about how similar participants' forehand stokes were to the “criterion model” was indicated by the movement criterion scores. Movement criterion was measured against a scoring scale in which a higher score indicated a more ideal movement form according to the “criterion model” of the forehand stroke typically taught by coaches. For both the specific and multiple target tasks, the LP group displayed significantly higher movement criterion scores at post and retention tests, providing evidence that the LP group performed more closely to the “criterion model”. On the other hand, the NP group only showed changes in movement criterion scores at post and retention tests for specific trials but not for multiple target trials, suggesting the presence of degeneracy. Participants from the NP group were able to adapt to the constraints of the tasks and adjust their hitting patterns accordingly. Furthermore, any changes in the forehand stroke for the NP group were different from what is typically deemed as the ideal forehand stroke, perhaps representing movement patterns that were more individualized.

Inter-individual cluster analysis provided a more in-depth understanding of the movement pattern changes that occurred following the 4-week intervention for specific target trials. As predicted, there were more movement clusters in the NP group (7 clusters) than in the LP group (3 clusters) at post-test, verifying that the presence of degeneracy is stronger in the NP group. Even though the NP group showed a greater variety of movement patterns as a result of more opportunities to explore, participants in this group improved their accuracy scores just as well as the LP group. This is consistent with previous studies [19], [30], [44] and provides evidence that there is more than one ideal way of hitting the ball to achieve the same task successfully.

An interesting observation was that the number of movement clusters for the LP group increased to 7 clusters at retention test. This greater variety of movement patterns at retention test coincided with the significant decrease in movement criterion scores from post to retention test for the multiple target trials, suggesting that learning the “criterion model” was not permanent. After a period without intervention, learners in the LP group seemed to incline towards a preferred stable state which was more appropriate for their individual composition. As learners in the LP group started to use movement patterns that they were more comfortable with, performance accuracy scores for the multiple target trials also improved during the retention test.

At post-test, the two more dominant clusters found in the LP group (i.e., C1 followed by C2) displayed characteristics that closely mimicked the given forehand cues in the LP instructions. For example, the racquet rotation angle was close to 0° at the start of the swing (preparation phase) indicating that the racquet head was pointing towards the net (Figure 4E), followed by an external rotation at backswing and an internal rotation of the right shoulder at the end of the swing (follow through) as the racquet ended above the left shoulder (Figure 3E). In accordance with traditional methods of skill acquisition, if the prescribed “criterion model” was the idealized motor pattern for performing the forehand stroke, all participants found in the dominant clusters should show better performance accuracy scores (see William & Hodges [3]). In terms of accuracy scores, two of the highest scoring LP participants (LP1 = 2.1 points; LP10 = 2.0 points) were found in C1 and C2 at post-test for specific target trials (Figure 5E). At the same time, the participants in the LP group who performed the worst (LP5 = 0.2 points; LP6 = 0.0 points; LP8 = 0.3 points) in terms of accuracy scores had trials that were also found predominantly in C1 and C2 (Figure 5E). This meant that even though these learners were able to perform the stroke in the “ideal” way, their performance accuracy scores showed otherwise. Furthermore, these three participants had among the highest scores for movement criterion scores (LP5 = 19; LP6 = 20; LP8 = 17) implying that their movement patterns were “almost perfect” or “perfect” in the case of LP6. LP6 was a typical example in which achieving the ideal movement technique, taught in a prescriptive and repetitive approach, resulted in a possible over conscious control of movement [45] or freezing of coordinative structures [46], leading to detrimental accuracy performance at post-test.

The NP participants on the other hand were not concerned with achieving the “right” movement pattern but displayed a greater variety of movement solutions putatively more functional for the individual learner. The highest scoring participant for performance accuracy in the specific target trials at post-test was NP17 (score = 2.0) found predominantly in C6 (Figure 5B). This participant had a movement crietrion score of 12 indicating that the movement patterns utilized were different from the “criterion model”. Visual inspection of C6 in Figure 3B and 4B showed that the movemement patterns of NP17 were quite distinct from the movement patterns of the other movement clusters found within the NP group as well as compared to the LP group (Figure 3E and 4E). Another participant, NP23, who also achieved among the highest performance accuracy scores (score = 1.4) in the NP group had trials found in six of the seven movement clusters, displaying distinct movement pattern characteristics from NP17 (Figure 5B). The presence of degeneracy is evident among these two highest scoring NP participants as different methods of achieving the same task goals were used by both participants and even within participant for the case of NP23. Research from an ecological dynamics perspective on skilled performance in sport has demonstrated the relationship between performance accuracy and functional variability, highlighting that degeneracy plays a functional role in helping performers to adapt to ever-changing task demands during practice and performance [21], [47], [48]. Further investigation showed that two participants from the NP group with the lowest accuracy scores (NP20 = 0.4; NP21 = 0.6) had trials spread among five of the seven clusters and were in transition from the coordination to control stage of learning. Similar to previous studies, perhaps this greater variability may represent exploratory behavior as the novice learners attempted to search for stable and functional states of coordination [40].

The results in this study show that movement variability is not necessarily detrimental but rather an essential process of acquiring a new skill. It provides new empirical evidence that there are multiple ways of achieving a task goal and challenges the common misconception that learners must achieve the “ideal” movement pattern to be successful. This study also supports that degeneracy is beneficial in learning a sports skill and verifies that this exploratory process is effectively facilitated by the NP approach [49]. In essence, the NP approach was able to cater for individual learning differences such that each child did not have to conform to a pre-determined movement pattern but were provided suitable boundaries to explore and discover unique functional movement solutions. Indeed, the NP approach prepares the individual with a variety of movement solutions to cope with a dynamic sporting environment.

From a practical viewpoint, practitioners should be less concerned if learners are not performing the “correct” movement pattern but emphasize instructions that focus on ensuring representativeness, establishing information-movement couplings, movement outcomes, functional movement variability and the manipulation of constraints so that learners can explore and discover effective movement solutions. While there may be concerns in the implementation of the NP approach such as ‘whether exploratory learning is time consuming’, ‘whether the practitioner has the right level of expertise and competency’, and ‘whether this approach is actually impactful’ [13], this study provides empirical evidence that NP is effective and is an instructional approach worth pursuing.

## Conclusions

While this study serves as a useful starting point to appreciate the potential benefits of NP at the individual level, further evidence and investigation is necessary. In particular, we signal the need for more representative learning designs such as that adopted in the present study to protect the practical significance of the research. Future studies should investigate how NP can be an optimal approach relative to training both in a game and team/class setting. What are the interactions that occur between teammates in terms of movement displacement? Will the dynamic interactions between other players and teammates escalate the ‘chaos’ present in a NP approach to a degree which contributes constructively to learners or is it destructive? How will NP affect motivation in a learning environment? There are potentially many logistical barriers for teachers to overcome in implementing NP amongst large groups of learners but in our opinion they are worth tackling and are not insurmountable. The road ahead may be challenging, but nevertheless this journey could help ensure individuals better prepare for a lifetime of physical activity and fulfilment from sports.
